# Non-steroidal anti-inflammatory drug delays corneal wound healing by reducing production of 12-hydroxyheptadecatrienoic acid, a ligand for leukotriene B_4_ receptor 2

**DOI:** 10.1038/s41598-017-13122-8

**Published:** 2017-10-16

**Authors:** Satoshi Iwamoto, Tomoaki Koga, Mai Ohba, Toshiaki Okuno, Masato Koike, Akira Murakami, Akira Matsuda, Takehiko Yokomizo

**Affiliations:** 1 Department of Biochemistry, Juntendo University School of Medicine, Tokyo, Japan; 2 Department of Ophthalmology, Juntendo University School of Medicine, Tokyo, Japan; 3 Priority Organization for Innovation and Excellence, Program for Leading Graduate Schools “HIGO (Health Life Science: Interdisciplinary and Glocal Oriented) Program”, Kumamoto University, Kumamoto, Japan; 4 Department of Cell Biology and Neuroscience, Juntendo University School of Medicine, Tokyo, Japan

## Abstract

Non-steroidal anti-inflammatory drugs (NSAIDs) are widely used to reduce inflammation by suppressing cyclooxygenases (COXs). NSAID eye drops are frequently prescribed after ocular surgery to reduce inflammation and pain, but this treatment has clinically significant side effects, including corneal ulcer and perforation. The molecular mechanisms underlying these side effects remain unknown. Recently, the COX product 12(*S*)-hydroxyheptadeca-5*Z*,8*E*,10*E*-trienoic acid (12-HHT) was identified as an endogenous ligand for leukotriene B_4_ receptor 2 (BLT2), which is important in maintenance of epithelial homeostasis. We hypothesized that NSAID-dependent corneal damage is caused by reduced production of 12-HHT. Diclofenac eye drops decreased the abundance of downstream products of COX and delayed corneal wound healing in BALB/c mice. Expression of BLT2 was observed in murine ocular tissues including cornea, and in human corneal epithelial cell line and human primary corneal epithelial cells. In BLT2-knockout mice, corneal wound healing was delayed, but the diclofenac-dependent delay in corneal wound healing disappeared. 12-HHT accelerated wound closure both in BLT2-transfected corneal cell line and human primary corneal epithelial cells. Thus, our results reveal that NSAIDs delay corneal wound healing by inhibiting 12-HHT production, and suggest that stimulation of the 12-HHT/BLT2 axis represents a novel therapeutic approach to corneal wound healing.

## Introduction

Leukotriene B_4_ receptor 2 (BLT2), a class A G-protein–coupled receptor (GPCR), was originally identified in our laboratory as a low-affinity receptor for leukotriene B_4_ (LTB_4_)^1^. In mice, BLT2 is expressed in small intestine, colon, and skin, whereas it is expressed ubiquitously in human^1,2^. We recently generated BLT2-deficient mice and used them to analyze the *in vivo* function of BLT2. Using BLT2-knockout (KO) mice, we identified the crucial roles of BLT2 in barrier function in intestine and skin^3,4^. Subsequently, we reported that BLT2 potentiates epithelial cell adhesion by up-regulating tight junctional proteins, including claudin 4^5^. Although LTB_4_ was assumed to be the ligand for BLT2, we recently identified 12(*S*)-hydroxyheptadeca-5*Z*,8*E*,10*E*-trienoic acid (12-HHT), a compound without known biological functions, as the endogenous ligand for BLT2^6^. 12-HHT is synthesized from arachidonic acid by cyclooxygenase (COXs) and/or thromboxane A_2_ synthase (TXA_2_S), and its production is suppressed by non-steroidal anti-inflammatory drugs (NSAIDs)^7^. Previous reports suggested that the 12-HHT/BLT2 axis plays important roles, especially in tissues exposed to the external environment, such as intestine and skin. Currently, however, the function of the 12-HHT/BLT2 axis in the eye, which also faces the external environment, remains unknown.

NSAID-containing eye drops are used clinically to suppress inflammation after intraocular surgery. Despite its importance and utility, topical eye application of NSAIDs can cause corneal epithelial injuries, ulceration, and perforation, and can also delay corneal epithelialization^8–10^. However, the molecular mechanisms by which NSAIDs delay corneal wound healing are unclear. Based on the observation of BLT2 expression in murine and human corneal epithelium, we hypothesized that delayed corneal wound healing by NSAIDs might be due to reduced production of 12-HHT and subsequent inhibition of BLT2 signaling. We investigated this possibility in this study.

## Results

### NSAIDs delay corneal wound healing

**Figure 1:**
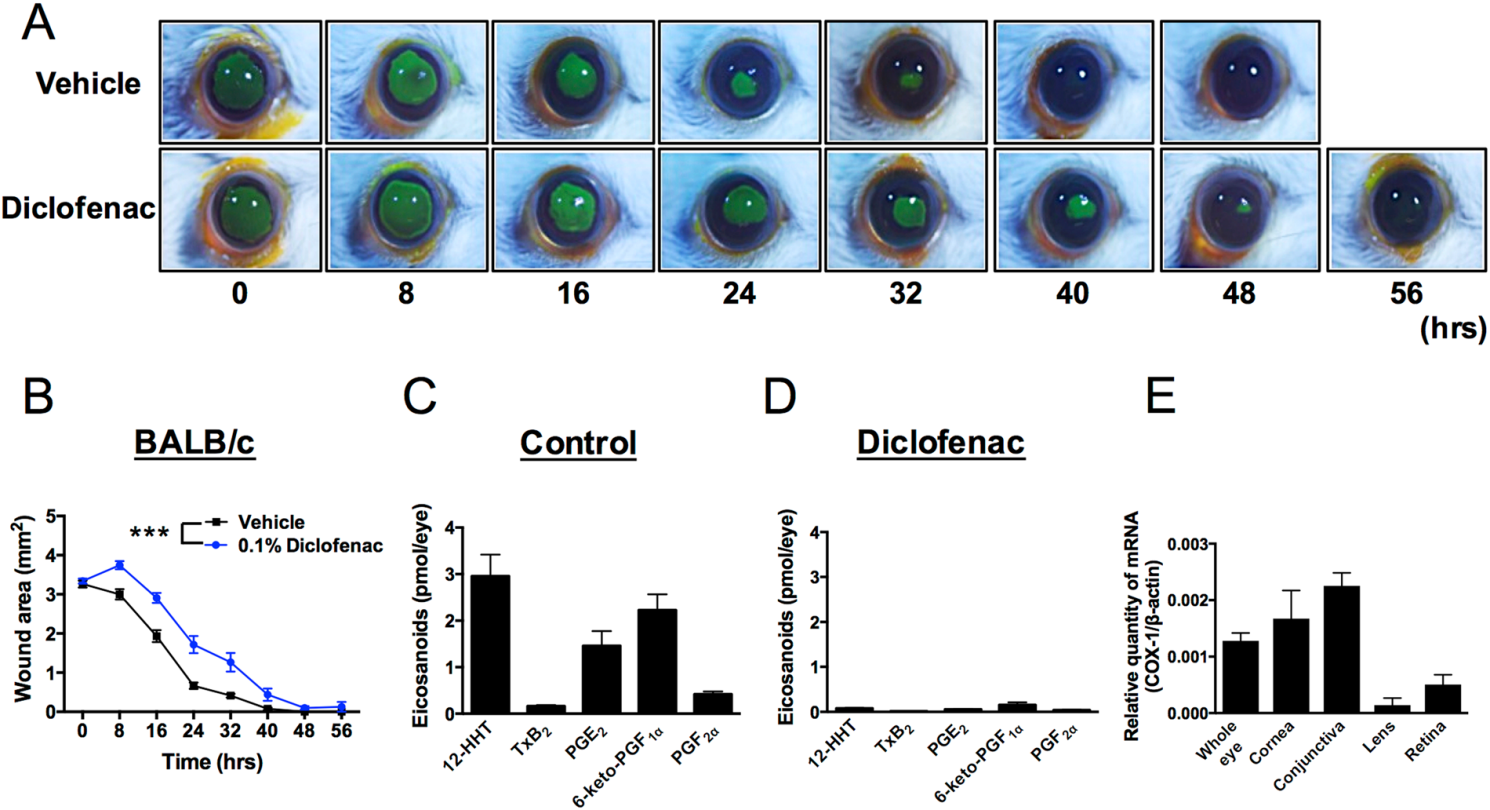
The NSAID diclofenac suppresses 12-HHT production and delays corneal wound healing in BALB/c mice. **(A)** Representative macroscopic images of fluorescein-stained corneal wounds in BALB/c mice after treatment with or without 0.1% diclofenac in PBS for 2 days (four times/day). **(B)** Wound area was measured every 8 hr after wounds were introduced in 13week old mice (n = 5–6). Error bars indicate means ± S.E. Data were analyzed by two-way ANOVA: ***p < 0.001. **(C**,**D)** Mice were treated with 0.1% diclofenac in PBS (Diclofenac) or vehicle control (Control) for 2 days (four times/day). Levels of eicosanoids and 12-HHT in the eye were quantified by LC-MS/MS (n = 3). Error bars indicate means ± S.E. **(E)** Expression of COX-1 mRNA in mouse cornea, conjunctiva, lens, and retina (n = 3).

Although corneal defects have been clinically reported as detrimental side effects of NSAIDs^8,9^, this phenomenon has not been explored experimentally in a detailed manner. To address this issue, we generated a murine model of corneal wound healing and used it to examine the effects of NSAIDs. In BALB/c mice, corneal wound area decreased in a time-dependent fashion, with complete wound closure occurring after 32 hr (Fig. 1A). To test the effects of NSAIDs in this system, the mice were treated with eye drops containing 0.1% diclofenac, almost the same concentration used in human patients in clinical settings, four times a day for 2 days. After diclofenac treatment, corneal epithelial wounds were introduced, and the fluorescein-stained wound area was measured every 8 hr until the wound was completely healed (Fig. 1A). As shown in Fig. 1A, corneal wound healing was slower in mice treated with diclofenac than in vehicle-treated mice. Quantification of the data confirmed that diclofenac significantly delayed corneal wound healing (Fig. 1B).

NSAIDs inhibit COX and suppress production of COX-dependent arachidonic acid metabolites, including prostaglandins (PGs), thromboxane A_2_, and 12-HHT^7^. To determine which metabolites are present in the eye, we performed lipidomics analyses, and also examined the effect of diclofenac treatment. We detected high levels of 12-HHT in murine eye, although the amount of TxB_2_ was quite low (Fig. 1C), suggesting that production of 12-HHT in the eye is independent of coagulation and TXA_2_S. In addition to 12-HHT, we also detected PGE_2_ and 6-keto-PGF_1_α (a stable metabolite of PGI_2_) (Fig. 1C), but leukotrienes including LTB_4_, LTC_4_, LTD_4_, and LTE_4_ were below the detection limit (data not shown). All eicosanoids detected in murine eye disappeared after treatment with diclofenac (Fig. 1D), and shunting to leukotrienes was not observed (data not shown). To elucidate which tissues produce PGs, TxA_2_, and 12-HHT, we next measured mRNA expression level of COX-1 in mouse eye tissues. As shown in Fig. 1E, cornea and conjunctiva dominantly expressed COX-1 compared with lens and retina, suggesting that cornea and conjunctiva are the main source for production of eicosanoids. These data suggest that NSAIDs delay corneal wound healing in mice, possibly due to the reduced production of eicosanoids in ocular surface tissues of the eye.

### BLT2 is expressed in corneal epithelial cells in mouse and human

**Figure 2:**
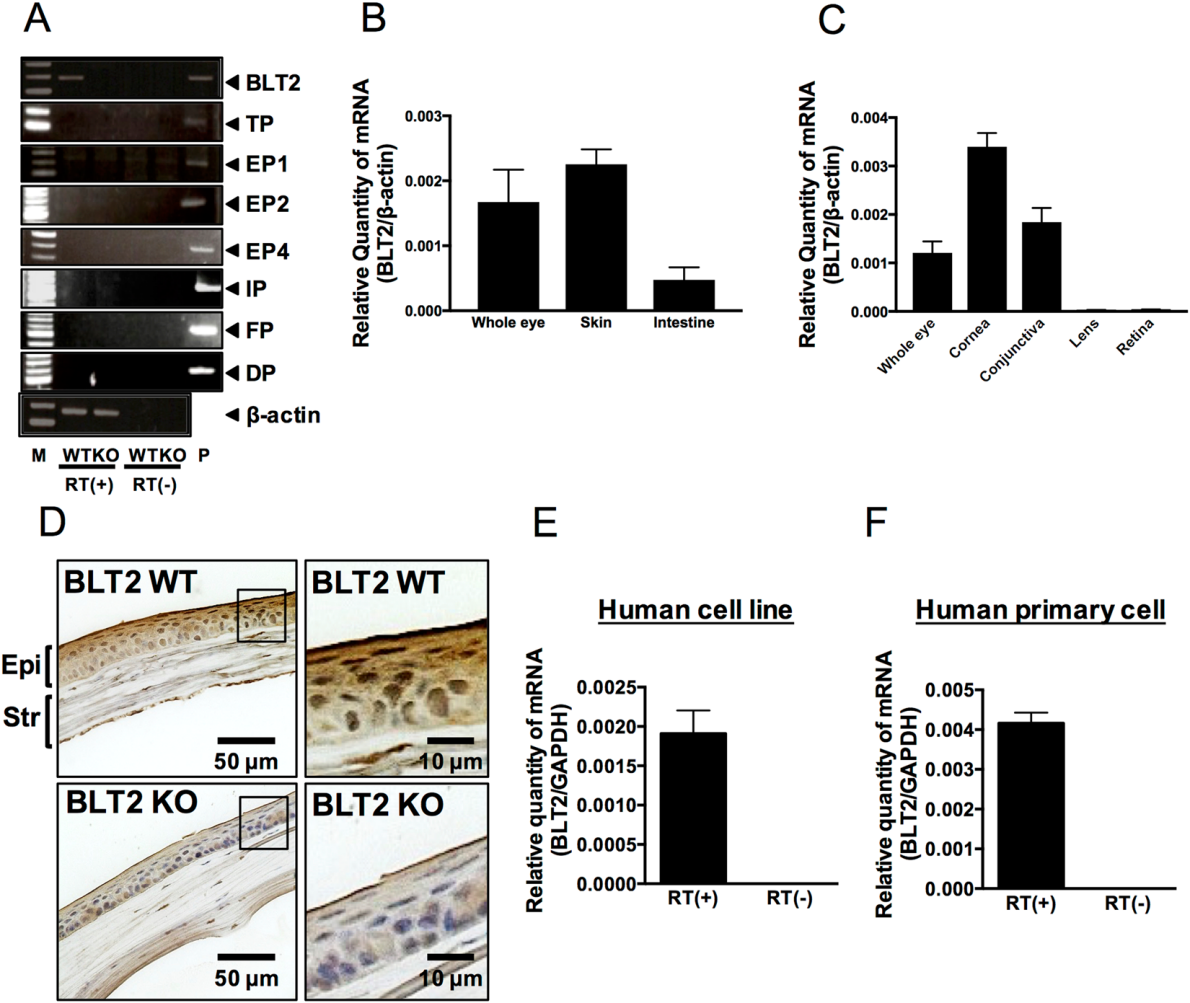
Expression of BLT2 in murine eye. **(A)** RT-PCR was performed to examine BLT2 and EP receptors in cornea. **(B,C)** Mouse *Blt2* mRNA expression was determined by qPCR analysis in mouse eye, skin, intestine **(B)**, cornea, conjunctiva, lens, and retina **(C)** (n = 3). β-actin was used as an internal control. **(D)** Immunohistochemical staining of BLT2 in cornea of BLT2 WT or BLT2 KO mice. (Magnification: 400X) **(E**,**F)** Expression levels of human *BLT2* mRNA were measured by qPCR in the human corneal epithelial cell line HCET **(E)** and primary human corneal epithelial cells **(F)**. *GAPDH* was used as an internal control. Error bars indicate means ± S.E. (n = 3). RT: reverse transcription. Str: stromal layer; Epi: epithelial layer of cornea.

Because we detected 12-HHT, TxB_2_, PGE_2_, 6-keto-PGF_1_α, and PGF_2_α in whole murine eye (Fig. 1C), we next performed reverse transcription–polymerase chain reaction (RT-PCR) to examine the corneal expression of eight receptors for those eicosanoids (*i.e*., BLT2, TP, EP1, EP2, EP4, IP, FP, and DP). Because BLT2 was the only receptor we detected in murine cornea (Fig. 2A), we compared the expression level of BLT2 in various tissues of BALB/c mice by quantitative real-time RT-PCR. The expression level of BLT2 in the eye was comparable to those in skin and intestine, the tissues in which BLT2 is most highly expressed (Fig. 2B). Both skin and intestine express the functional 12-HHT/BLT2 axis both *in vitro* and *in vivo* ^2–6^. Interestingly, BLT2 expression was observed only in parts of the eye associated with the ocular surface, including the cornea and conjunctiva, but not in the lens and retina (Fig. 2C). Immunohistochemical analysis showed that BLT2 protein was present in the epithelial layer in wild-type (WT) mouse cornea, but was absent from BLT2 KO mouse cornea (Fig. 2D). BLT2 signal was also detected in corneal endothelial cells (not shown), which is consistent with our previous findings in lung endothelial cells^11^. We also detected expression of BLT2 in the human corneal epithelial cell line HCET (Fig. 2E), as well as in primary human corneal epithelial cells (Fig. 2F). No signals were detected in negative control samples lacking reverse transcriptase (Fig. 2E and F). Taken together, these findings demonstrate that BLT2 is expressed in corneal epithelial cells both in mouse and human.

### WT and BLT2 KO cornea are not histologically different

**Figure 3:**
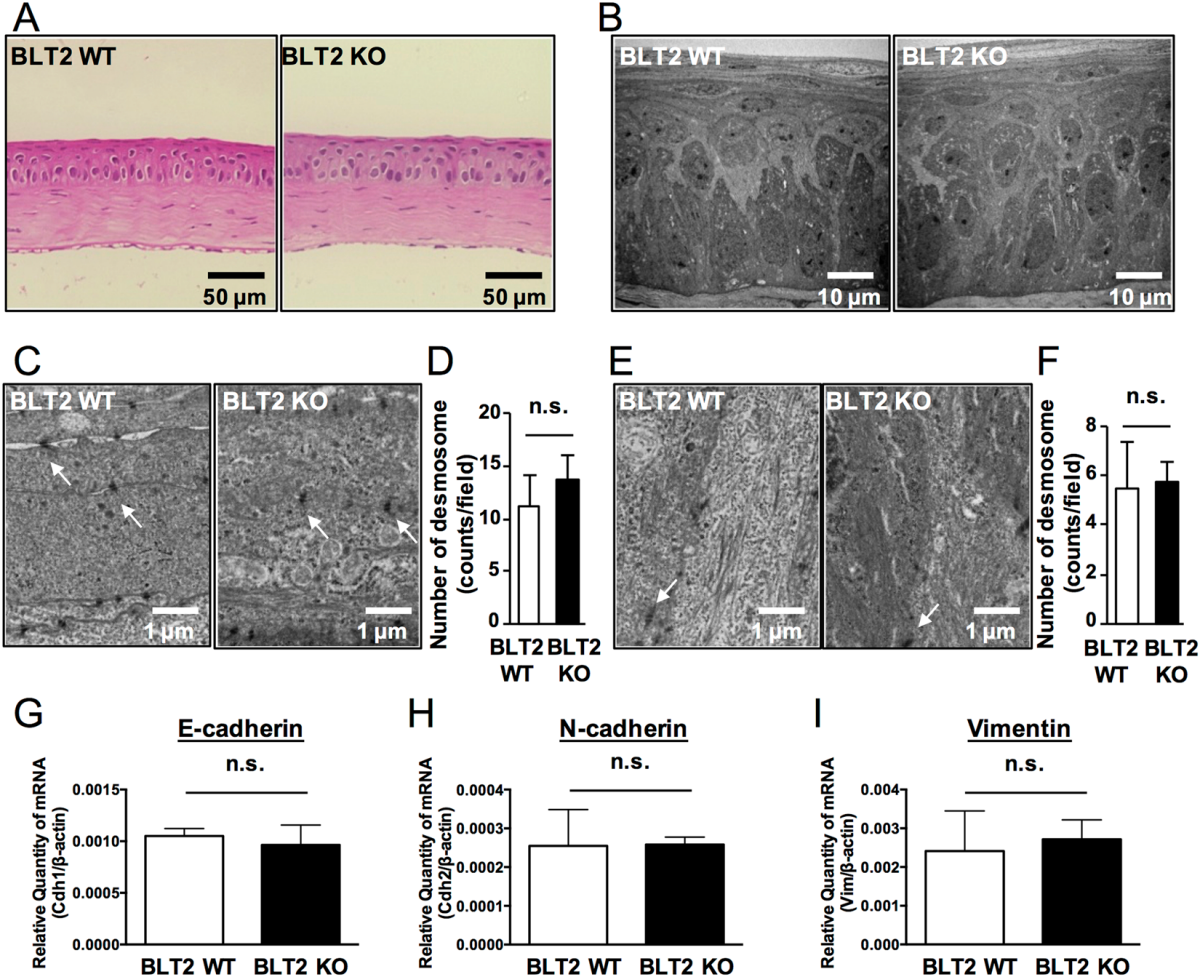
BLT2 KO cornea has no overt phenotype at steady state. **(A)** Representative HE-stained sections of cornea from BLT2 WT (*left panel*) and BLT2 KO mice (*right panel*). **(B)** Electron micrographs of cornea from BLT2 WT (*left panel*) and BLT2 KO mice (*right panel*). **(C**,**E)** Representative electron micrographs of superficial layer **(C)** and basal layer **(E)** in BLT2 WT (*left panel*) and BLT2 KO cornea (*right panel*). Arrows indicate desmosomes. **(D**,**F)** Quantification of the number of desmosomes in the superficial layer **(E)** and basal layer **(F)**. **(G**–**I)** mRNA expression of mouse E-cadherin (*Cdh1*), N-cadherin (*Cdh2*), and vimentin (*Vim*) was determined by qPCR analysis of mouse cornea. Error bars indicate means ± S.E. (n = 3). n.s.: not significant. Scale bars: 50 µm (**A**), 10 µm (**B**), 1 µm (**C**,**E**).

We next examined steady-state corneal phenotypes in BLT2 WT and BLT2 KO mice. HE-stained cornea sections did not overtly differ between the two genotypes (Fig. 3A), and electron-microscopic examination of the cornea (Fig. 3B) confirmed this finding. The cornea is divided into three layers: epithelium, stroma, and endothelium. The epithelial layer, in turn, consists of three types of cells: basal, winged, and superficial. Because desmosomes in the various epithelial cell layers provide structural stability and contribute to the barrier function of the corneal epithelium, we next counted the desmosomes in the superficial (Fig. 3C and D) and basal layers (Fig. 3E and F). The numbers of desmosomes in the superficial and basal layers were comparable between BLT2 WT and BLT2 KO mice (Fig. 3C–F). Furthermore, we measured the levels of mRNAs encoding adhesion proteins (E-cadherin, N-cadherin, and Vimentin) in BLT2 WT and BLT2 KO mice, and found that the levels of all three were comparable between genotypes (Fig. 3G–I). These data demonstrate that, at steady state, BLT2 WT and BLT2 KO cornea do not overtly differ at the histological level.

### 12-HHT/BLT2 axis promotes corneal wound healing *in vivo*

**Figure 4:**
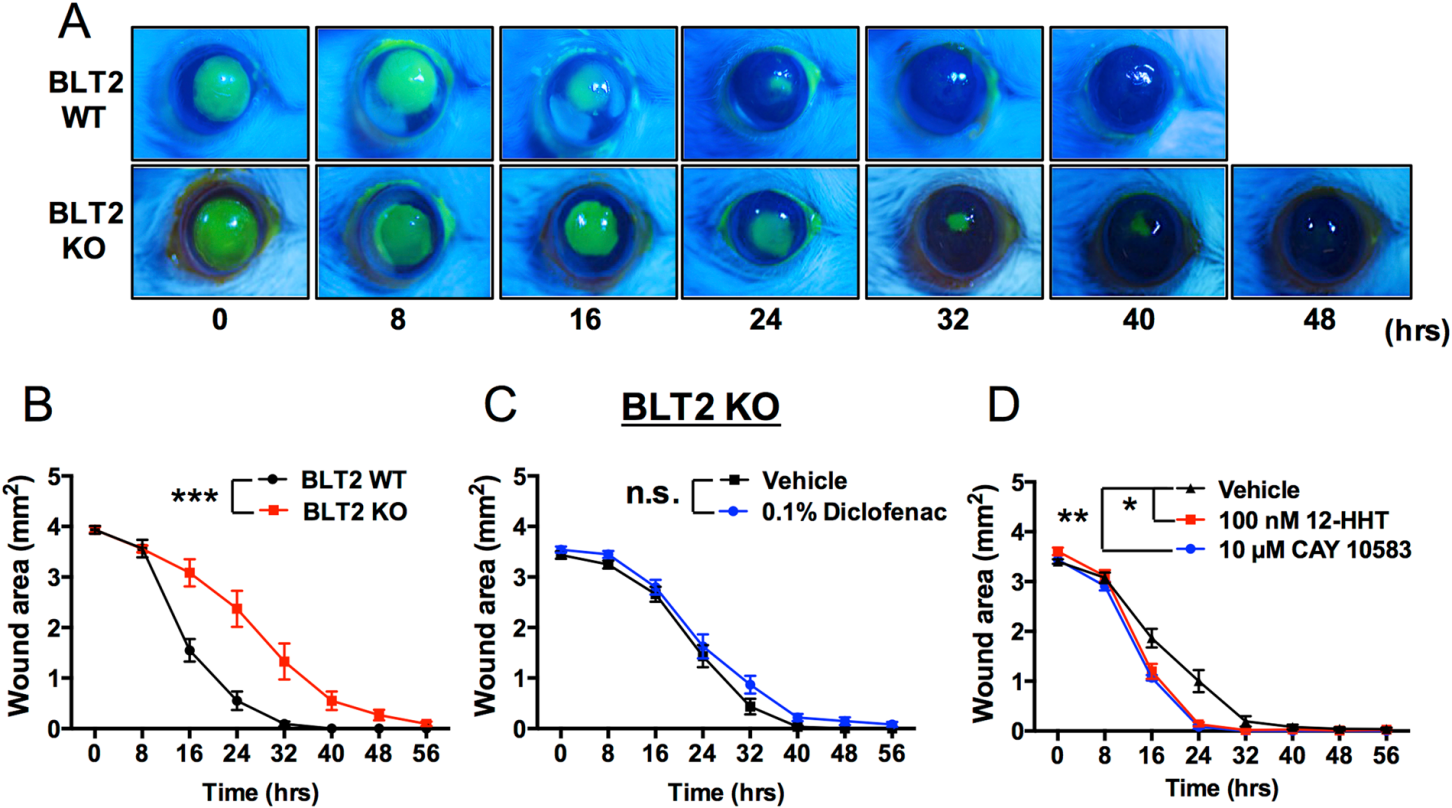
Corneal wound healing is delayed in BLT2 KO mice. **(A)** Representative macroscopic images of fluorescein-stained corneal wounds in BLT2 WT and BLT2 KO mice at the indicated times after wounding. **(B)** Corneal wound area was measured in 11–12 weeks old BLT2 WT and BLT2 KO mice. Data were analyzed by two-way ANOVA: ***p < 0.001. (n = 8–12) **(C)** Corneal wounds were measured in BLT2 KO mice after treatment with 0.1% diclofenac for 2 days. Data were analyzed by two-way ANOVA; n.s.: not significant. **(D)** Corneal wounds were measured in 10 week old WT mice after treatment with 0.1% diclofenac for 2 days and treatment of 100 nM 12-HHT or 10 µM CAY10583 (BLT2 agonist). Data were analyzed by two-way ANOVA (8–32 hour); *p < 0.05 **p < 0.01 (n = 5–6).

Next, we examined the effects of the 12-HHT/BLT2 axis on corneal wound healing in BLT2 WT and BLT2 KO mice. Defects in the cornea were visualized by fluorescein staining, and the fluorescein-positive area was quantified as the wound area (Fig. 4A). Corneal wound healing was clearly delayed in BLT2 KO mice in comparison with BLT2 WT mice (Fig. 4B). However, the diclofenac-induced delay in corneal wound healing was abolished by BLT2 deficiency (Fig. 4C). Furthermore, BLT2 ligands including 12-HHT and CAY10583 significantly accelerated corneal wound healing under the diclofenac-treated condition (Fig. 4D). These results demonstrate that BLT2 promotes corneal wound healing, and that the NSAID-dependent delay in corneal wound healing is caused by reduction of 12-HHT, a ligand for BLT2 receptor.

### The 12-HHT/BLT2 axis accelerates corneal epithelial cell migration *in vitro*

**Figure 5:**
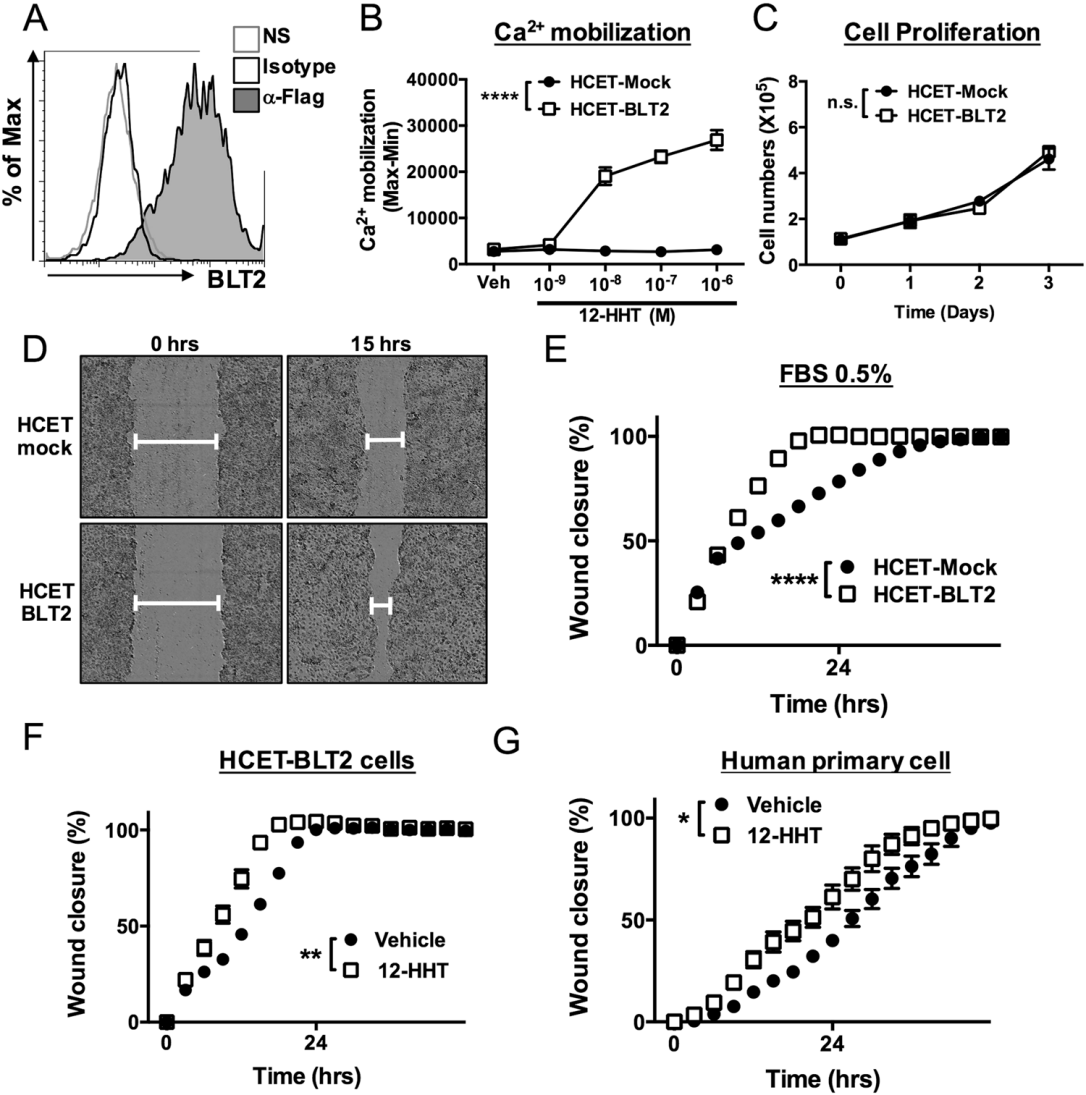
12-HHT/BLT2 signaling accelerates wound closure in corneal epithelial cells *in vitro*. **(A)** Flow cytometric analysis of HCET cells stably expressing FLAG-tagged human BLT2. NS: no staining. **(B)** 12-HHT–dependent Ca^2+^ mobilization was assessed after loading of Fluo-8 AM in BLT2-overexpressing HCET cells. Error bars indicate means ± S.E. (n = 4). **(C)** Cell growth of HCET-Mock and HCET-BLT2 (n = 3). **(D)** Representative fields of HCET-Mock (*upper*) and HCET-BLT2 cells (*bottom*) at 0 and 15 hr after scratching. **(E)** HCET-Mock and HCET-BLT2 cells were subjected to scratch assay in medium containing 0.5% FBS. Wound area was measured and is shown as wound closure rate (%). **(F)** HCET cells were subjected to scratch assay in the presence of 1 µM 12-HHT. **(G)** Primary human corneal epithelial cells were subjected to scratch assay. 12-HHT (10 nM) was added, and the wound closure rate was measured. All data represent means ± S.E. Data were analyzed by two-way ANOVA: *p < 0.05, **p < 0.01, ****p < 0.0001; n.s.: not significant.

To investigate the mechanism by which 12-HHT/BLT2 accelerates corneal wound healing, we generated human corneal HCET cells stably overexpressing FLAG-tagged BLT2. Surface expression of BLT2 was confirmed by flow cytometric analysis after staining of the FLAG tag (Fig. 5A). To confirm functional expression of BLT2 in these cells, we performed Ca^2+^ mobilization assays. The BLT2 ligand 12-HHT increased Ca^2+^ mobilization in a dose-dependent manner only in HCET-BLT2 cells, but not in HCET-Mock cells (Fig. 5B). To determine whether BLT2 promotes corneal epithelial cell proliferation and/or cell migration, we counted the cells. As shown in Fig. 5C, cell proliferation was comparable between HCET-BLT2 and HCET-Mock cells. To investigate the contribution of BLT2 to corneal epithelial cell migration, we performed scratch assays in BLT2-overexpressing HCET cells. As shown in Fig. 5D and E, *in vitro* wound closure was faster in HCET-BLT2 cells than in HCET-Mock cells in the medium containing 0.5% FBS (corresponding to 1–3 nM 12-HHT, which can activate BLT2 receptor) (Fig. 5D and E). In addition, 12-HHT accelerated cell migration in HCET-BLT2 cells (Fig. 5F). Furthermore, we investigated the effect of 12-HHT stimulation on migration of primary human corneal epithelial cells. Consistent with the results obtained in BLT2-overexpressing HCET cells, wound closure was accelerated by 12-HHT in primary corneal epithelial cells (Fig. 5G). Collectively, these findings indicate that the 12-HHT/BLT2 axis promotes corneal wound healing by accelerating the migration of corneal epithelial cells.

## Discussion

**Figure 6:**
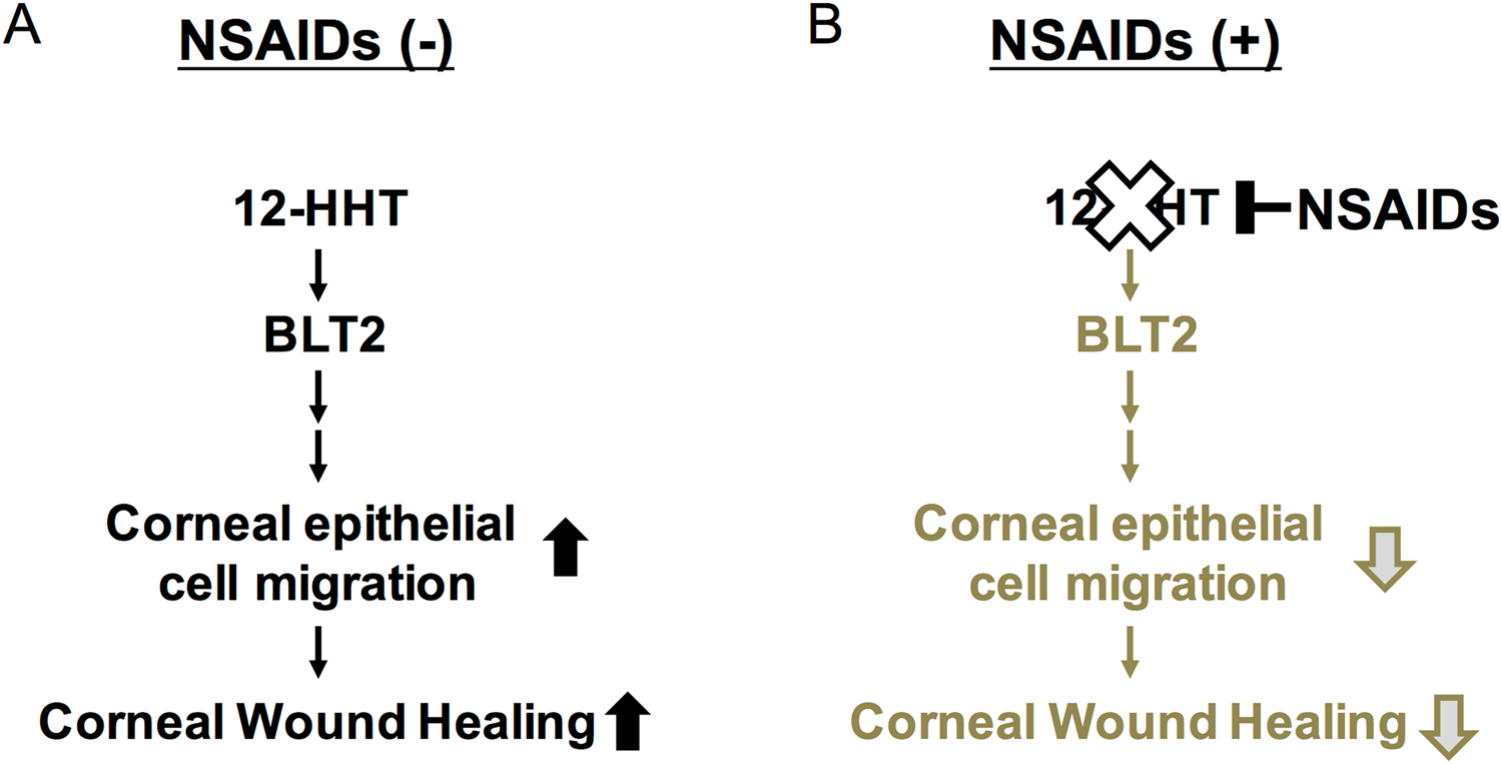
Schematic illustration of how the 12-HHT/BLT2 signaling pathway functions in corneal wound healing, and how NSAIDs delay healing. **(A)** 12-HHT activates BLT2 signaling and subsequently accelerates corneal epithelial cell migration, resulting in healing of corneal wounds. **(B)** NSAID eye drops inhibit production of 12-HHT in the eye, resulting in suppression of downstream events and ultimately delaying the corneal wound healing process.

This study provides the first elucidation of the molecular mechanisms by which NSAIDs delay corneal wound healing *in vitro* and *in vivo* (Fig. 6). NSAIDs inhibited the production of 12-HHT, a lipid mediator that activates BLT2 receptor and thereby accelerates corneal epithelial cell migration *in vitro* and *in vivo*. This is the first report of the role of BLT2 in murine and human eyes. Although there are some reports suggesting the expression of EP receptors in the human cornea^12,13^, we failed to detect EP1, EP2, and EP4 receptors in mouse cornea by semi-quantitative RT-PCR (Fig. 2A). This discrepancy might be due to the different assays, because previous reports used immunohistochemistry to detect EP receptors. Of course, we cannot exclude the possible contribution of EP receptors for corneal function. We believe that 12-HHT/BLT2 axis is critically involved in NSAIDs-dependent delay of corneal wound healing. Moreover, although we did not investigate the downstream target molecules of 12-HHT/BLT2–dependent corneal epithelial cell migration in this study, our results suggest that the 12-HHT/BLT2 axis represents a novel therapeutic target for treatment of corneal damage. Autologous serum eye drops, but not plasma, are used to improve persistent corneal epithelial defects in the clinic^14–16^. However, the mechanism by which serum eye drops accelerate corneal wound healing is not known. Because blood coagulation greatly increases the level of 12-HHT, serum contains a much higher concentration of 12-HHT than plasma. Our lipidomics analysis revealed that 12-HHT concentrations reach 900 nM and 300 nM in human and mouse serum, respectively^7^. Thus, our data partly explain how serum eye drops ameliorate corneal epithelial defects. We previously investigated the physiological roles of BLT2 using BLT2-deficient mice^17,18^, and showed that BLT2 plays important roles in tissues facing the external space, including intestine, skin, and lung^2–5,11^. Because the cornea is also exposed to the external environment, our data emphasized the crucial role of the 12-HHT/BLT2 axis as a regulator of surface barriers at host/environmental boundaries.

A particular goal of the present study was to reveal the molecular mechanisms underlying the NSAID-dependent delay in corneal wound healing. NSAID eye drops are frequently used to suppress ocular inflammation. Despite their utility in the clinical setting, NSAIDs can cause corneal epithelial injuries, ulceration, and perforation, and also delay of corneal re-epithelialization^8–10^. These adverse effects are serious clinical problems that must be resolved; however, the underlying molecular mechanisms by which NSAIDs delay corneal wound healing remain elusive. The results of this study provide an answer to this longstanding question. We showed that the 12-HHT/BLT2 axis promotes corneal wound healing *in vitro* and *in vivo*. 12-HHT is an endogenous ligand for BLT2, as we showed previously^6^, and its production is dependent on COX and/or TXA_2_S^7^. Because 12-HHT is produced by COX, NSAIDs inhibit its production^3,7^. Therefore, it is possible that NSAID-induced corneal dysfunctions, such as ulceration, perforation, and delay of wound healing, are due to inhibition of COX-dependent 12-HHT production, which subsequently suppresses the BLT2-dependent corneal epithelial cell migration. These data provide novel insights into the optimal use of NSAIDs in various eye diseases, with respect to corneal wounds and the amount of 12-HHT.

In addition, our results revealed the critical role of the COX-dependent lipid mediator 12-HHT in corneal epithelial wound healing. Previous reports showed that Lipoxin A_4_ (LXA_4_) and neuroprotectin D1 (NPD1), but not pro-inflammatory lipid mediators such as LTB_4_ and 12*R*-hydroxyeicosatrienoic acid, play important roles in corneal wound healing by recruiting neutrophils^19–21^. Lipid mediators such as LXA_4_ and NPD1 are produced by 12/15-lipoxygenase–dependent pathways, which are not inhibited by NSAIDs. Corneal wound healing is a multi-step process that includes cell proliferation, migration, differentiation, stratification, and desquamation/apoptosis^22,23^. Therefore, to efficiently cure corneal wounds, multiple lipid mediators are required. Accordingly, in future work we plan to investigate the role of other lipid mediators, including lysophospholipids, sphingosine 1-phosphate (S1P), and pro-resolving lipid mediators^24–27^.

In summary, our results provide evidence that the 12-HHT/BLT2 axis promotes corneal epithelial cell migration, and that inhibition of 12-HHT production by NSAIDs delays corneal wound healing *in vitro* and *in vivo*. These findings resolve a longstanding problem in ophthalmology, and suggest a novel therapeutic target for corneal damage.

## Methods

### Mice

BLT2 KO mice were generated as previously described, and backcrossed with BALB/c mice for more than 12 generations^4^. Sex matched BLT2 KO and BLT2 WT mice, 11–13 weeks old, were used for the experiments. For the diclofenac treatment experiments, 10–12 weeks old female BALB/c mice were purchased from Charles River Inc (Shizuoka, Japan). All animals were reared under specific pathogen–free conditions and provided with food and water *ad libitum*.

### Ethics statement

All animal experiments were approved by the Ethical Committee for Animal Experiments in Juntendo University. All methods in this manuscript were carried out in accordance with the approved guidelines and regulations.

### Real-time PCR

Tissue samples, including skin, intestine, eye, cornea, conjunctiva, lens, and retina, were collected from five mice (10 eyes, *i.e*., two eyes/sample), and total RNA was extracted using the RNeasy Fibrous Tissue Kit (Qiagen) according to the manufacturer’s instructions. Total RNA (1 µg) was subjected to reverse transcription using the QuantiTect Reverse Transcription Kit (Qiagen) with random and oligo-dT mixed primers. For the human corneal epithelial cell line and primary human corneal epithelial cells, total RNA was extracted from 1 × 10^6^ cells using the Trizol reagent (Life Technologies). Total RNA (1 µg) was subjected to reverse transcription using the same kit. Target genes were detected by real-time PCR using Fast SYBR Green Master Mix (Applied Biosystems) on an ABI7500 Real-Time PCR system (Life Technologies). Semi-quantitative PCR was performed by using KOD -Plus- (TOYOBO). Condition of PCR was as follows: mouse BLT2 and mouse β-actin; 92 °C 2 min, 35cycles of 94 °C 48 sec, 60 °C 42 sec, 68 °C 30 sec and 68 °C 10 min, mouse DP; 94 °C 2 min, 35cycles of 94 °C 48 sec, 62 °C 42 sec, 68 °C 48 sec and 68 °C 10 min, mouse IP and mouse FP; 94 C° 2 min, 35cycles of 94 °C 48 sec, 62 °C 42 sec, 68 °C 59 sec and 68 °C 10 min, mouse EP1; 94 °C 2 min, 35cycles of 94 C° 48 sec, 65 °C 42 sec, 68 °C 48 sec and 68 °C 10 min, mouse EP2; 94 °C 2 min, 35cycles of 94 °C 48 sec, 65 °C 42 sec, 68 °C 48 sec and 68 °C 10 min, mouse EP4; 94 °C 2 min, 35cycles of 94 °C 48 sec, 64 °C 42 sec, 68 °C 25 sec and 68 °C 10 min, mouse TP; 94 °C 2 min, 35cycles of 94 °C 45 sec, 64 °C 42 sec, 68 °C 90 sec and 65 °C 10 min.

Primer sequences were as follows: human BLT2 (*LTB4R2*) Fw, 5′-TACCACGCAGTCAACCTTCTG-3′; human BLT2 Rv, 5′-GCGGTGAAGACGTAGAGCAC-3′; human GAPDH Fw, 5′-TGCCCTCAACGACCACTTTG-3′; human GAPDH Rv, 5′-CTCTTCCTCTTGTGCTCTTGCTG-3′; mouse cadherin 1 (*Cdh1*) Fw, 5′-CAGGTCTCCTCATGGCTTTGC-3′, mouse cadherin 1 (*Cdh1*) Rv, 5′-CTTCCGAAAAGAAGGCTGTCC-3′; mouse cadherin 2 (*Cdh2*) Fw, 5′-ATAGCCCGGTTTCACTTGAGA-3′, mouse cadherin 2 (*Cdh2*) Rv, 5′-CAGGCTTTGATCCCTCTGGA-3′; mouse vimentin (*Vim*) Fw, 5′-CGGCTGCGAGAGAAATTGC-3′, mouse vimentin (*Vim*) Rv, 5′-CCACTTTCCGTTCAAGGTCAAG-3′; mouse BLT2 (*Ltb4r2*) Fw, 5′- ACAGCCTTGGCTTTCTTCAG-3′; mouse BLT2 Rv, 5′-TGCCCCATTACTTTCAGCTT-3′; human BLT2 (*LTB4R2*) Fw, 5′-TACCACGCAGTCAACCTTCTG-3′; human BLT2 Rv, 5′-GCGGTGAAGACGTAGAGCAC-3′; mouse β-actin (*Actb*) Fw, 5′-CATCCGTAAAGACCTCTATGCCAAC-3′; mouse β-actin (*Actb*) Rv, 5′-ATGGAGCCACCGATCCACA-3′; mouse EP1 (*Ptger1*) Fw, 5′-CGTCGCTCTCGACGATTCCGAAAGACCGCA-3′, mouse EP1 (*Ptger1*) RV, 5′-CGATGGCCAACACCACCAACACCAGCAGGG-3′; mouse EP2 (*Ptger2*) Fw, TTCATATTCAAGAAACCAGACCCTGGTGGC-3′, mouse EP2 (*Ptger2*) Rv, 5′-AGGGAAGAGGTTTCATCCATGTAGGCAAAG-3′; mouse EP4 (*Ptger4*) Fw, 5′-TTCCGCTCGTGGTGCGAGTGTTC-3′, mouse EP4 (*Ptger4*) Rv, 5′-GAGGTGGTGTCTGCTTGGGTACG-3′; mouse TP (*TbxA2r*) Fw, 5′-TGCCTTGTTGGACTGGCGAGCCACTGACCC-3′, mouse TP (*TbxA2r*) Rv, 5′-CAGGTAGATGAGCAGCTGGTGCTCTGTGGC-3′; mouse IP (*Ptgir)* Fw, 5′-CCTGCAGTGTTTGTGGCCTATGCTCGAAAC-3′, mouse IP (*Ptgir)* Rv, 5′-CTGCTGTCTGGGGCGATGGCCTGAGTGAAG-3′; mouse DP (*Ptgdr)* Fw, 5′-AAAGGAACTGCTGCCTGCCTCAGGCAATCA-3′ mouse DP (*Ptgdr)* Rv, 5′-GTTCTCAAGTTTAAAGGCTCCATAGTACGC-3′; mouse FP (*Ptgfr)* Fw, 5′-GCATAGCTGTCTTTGTATATGCTTGTGATA-3′, mouse FP (*Ptgfr)* Rv, 5′-GTGTCGTTTCACAGGTCACTGGGGAATTAT-3′.

### Quantification of eicosanoids in mouse whole eyes

Eyes were collected after perfusion of mice with 50 ml of saline containing 4 U/ml heparin (FUSO, Pharmaceutical Industries, Japan) *via* the left ventricle. Lipids were extracted from two eyes with methanol containing deuterium-labeled internal standards. Each sample was diluted with water to yield a final methanol concentration of 20%, and then loaded on Oasis HLB cartridges (Waters). The column was subsequently washed with petroleum ether and water containing 0.1% formic acid. The samples were eluted with 200 µl of methanol containing 0.1% formic acid. Eicosanoids in each sample were quantified by LC-MS/MS as described previously^3,7^. LC-MS/MS analyses were performed by using a Shimadzu liquid chromatography system and tandem-connected a TSQ Quantum Ultra triple quadrupole mass spectrometer equipped with an electrospray ionization system (Thermo Fisher Scientific). Each sample was injected into the trap column, an Opti-Guard Mini C18 and concentrated sample was analyzed with an analytical column, a Capcell Pak C18 MGS3 (Shiseido, Tokyo, Japan). Separation of lipids was achieved by a step gradient with 0.1% formic acid in water and 0.1% formic acid in acetonitrile. The LC column eluent was introduced directly into a TSQ Quantum Ultra. All compounds were analyzed in a negative ion polarity mode. 12-HHT and eicosanoids were quantified by selective reaction monitoring (SRM); *m/z* 279 (parental ion MS) → 179 (daughter ion MS) for 12-HHT. A mixture of deuterium-labeled eicosanoids was used as internal standards. Because deuterium-labeled 12-HHT is not commercially available, 12-HHT was calibrated with deuterium-labeled LTB_4_. Automated peak detection, calibration, and calculation were carried out by using the XCalibur 1.2 software package.

### Histological analysis

Eyes were fixed in 10% formalin, embedded in paraffin, sectioned (10 µm thickness), and stained with hematoxylin and eosin (HE). For immunohistochemical analysis, antigen retrieval was performed by boiling the sample for 10 min in citrate buffer (pH 6.0). Primary antibody was a rabbit anti-mouse BLT2 polyclonal^3,11^ generated in our laboratory. Secondary antibody was biotin-conjugated anti-rabbit IgG, and streptavidin-conjugated horseradish peroxidase was used for the third reaction. Signals were visualized with 3,3′-diaminobenzidine (DAB). For electron microscopy, mice were euthanized, and the eyes were enucleated, fixed by immersion in 2.5% glutaraldehyde buffered with 0.1 M phosphate buffer, postfixed with 1% OsO_4_, dehydrated with a graded series of ethanol, and embedded in epoxy resin. Ultrathin sections were cut with an ultramicrotome (UC7; Leica microsystems, Vienna, Austria), stained with uranyl acetate and lead citrate, and observed with a Hitachi HT7700 electron microscope. To quantify the number of desmosomes, electron micrographs of superficial and basal layers of the cornea were randomly taken with a primary magnification of 5,000x. Data are expressed as the average number of desmosomes in three electron micrographs.

### *In vivo* wound healing analysis

The mouse model of corneal wound injury was generated as described previously^28^. Briefly, the day before the wound was made, 1% atropine eye drops (Nitten, Japan) were applied to avoid damage to the iris. After local anesthesia with oxybuprocaine hydrochloride (Santen Pharmaceutical, Japan), a trephine was used to introduce a 2 mm diameter wound in the cornea of the right eye. Epithelial defects were created using a spatula (INAMI Inc.). Each corneal wound was visualized with 1% fluorescein and monitored after surgery every 8 hr until the wound was completely healed. After wounding, levofloxacin eye drops (antibiotics, Santen Pharmaceutical, Japan) were used to prevent corneal infection. The area of the epithelial defect was measured using the ImageJ software. For diclofenac treatment, diclofenac was dissolved into PBS at a concentration of 0.1% (w/v). Diclofenac administration (4 times/day) started 2 days before the wound was made.

### Cell culture

The human corneal epithelial cell line HCET was maintained in DMEM/F12 (Wako Pure Chemical Industries), 10% FBS (Gibco), and 1% P/S (Nacalai Tesque). Primary human corneal epithelial cells were purchased from KURABO LTD (Bio-medical Department, Kurabo Industries LTD., Tokyo, Japan) and maintained in OcuLife^®^ BM supplemented with growth factors (KURABO).

### Generation of HCET cells overexpressing FLAG-tagged human BLT2

HCET cells were plated on 10 cm dishes 1 day before transfection. Cells at 60% confluence were transfected with pCXN2.1-FLAG-human BLT2 (hBLT2) plasmid or pCXN2.1 (Mock)^29^ using FuGENE^®^ HD. Forty-eight hours after transfection, medium was replaced with selection medium [DMEM/F12 (1:1), 10% FBS, 1% P/S, 0.15 mg/ml G418], and the cells were cultured for an additional 4 weeks. Cells were collected and resuspended in FACS buffer (PBS + 2% FBS), incubated with 1 µg/ml anti-FLAG antibody 2H8^30^ at 4 °C for 60 min, and then stained with 1 µg/ml Alexa Fluor 488–conjugated goat anti-mouse IgG (Life Technologies) at 4 °C for 30 min. To isolate BLT2-expressing cells, the cells were sorted three times on a MoFlo (Beckman Coulter).

### *In vitro* scratch assay

Primary human corneal epithelial cells (1 × 10^4^ cells/well) and HCET cells overexpressing FLAG-hBLT2 (1 × 10^4^ cells/well) were plated onto collagen-coated 96-well ImageLock tissue culture plates (Essen Bioscience) and cultured for 48 hr. Wounds were made with a 96-well WoundMaker (Essen Bioscience). The wounded wells were washed twice with medium and treated with 100 µl of medium containing 12-HHT. Images were acquired automatically every 3 hr until the wound was completely closed (48 hr). In wound healing assays, cells were starved for 3 hr in 0.1% BSA containing medium before treatment with 12-HHT.

### Ca^2+^ mobilization assay

HCET-Mock and HCET-BLT2 cells (4 × 10^4^ cells/well) were seeded in glass-bottom 96-well plates and incubated for 24 hr. Cells were loaded with 5 µM Fluo-8 AM (AAT Bioquest, Inc.) in HBSS (pH 7.4) buffer containing 2.5 mM probenecid and 20 mM HEPES (pH 7.4) at 37 °C for 30 min, and further incubated at room temperature for 30 min. After two washes with buffer, the cells were stimulated with HBSS containing various concentrations of ligand. Agonist-induced intracellular calcium mobilization was determined using a FLEX Station (Molecular Devices, Sunnyvale, CA, USA).

### Statistical analysis

All data are expressed as mean ± SEM. Statistical significance was evaluated by Student’s *t*-test or ANOVA. All statistics were calculated using Prism 6 software (GraphPad Software).
